# Bimodal multispectral imaging system with cloud-based machine learning algorithm for real-time screening and detection of oral potentially malignant lesions and biopsy guidance

**DOI:** 10.1117/1.JBO.26.8.086003

**Published:** 2021-08-16

**Authors:** Narayanan Subhash, Suresh Anand, Ranimol Prasanna, Sandeep P. Managoli, Rinoy Suvarnadas, Vidyarani Shyamsundar, Karthika Nagarajan, Sourav K. Mishra, Migi Johnson, Mahesh Dathurao Ramanand, Sanjay C. Jogigowda, Vishal Rao, Kodaganur S. Gopinath

**Affiliations:** a Sascan Meditech Pvt Ltd, TIMed, Sree Chitra Tirunal Institute for Medical Science & Technology (SCTIMST), Thiruvananthapuram, Kerala, India; b Sree Balaji Dental College & Hospital, Center for Oral Cancer Prevention Awareness and Research, Chennai, Tamil Nadu, India; c Institute of Medical Sciences and SUM Hospital, Department of Oncology, Bhubaneswar, Orissa, India; d Government Dental College, Department of Oral Medicine and Radiology, Kottayam, Kerala, India; e Dayananda Sagar College of Dental Sciences, Department of Oral Medicine, Bangalore, Karnataka, India; f JSS Dental College & Hospital, Department of Oral Medicine, Mysore, Karnataka, India; g HCG Cancer Center, HCG Towers, Bengaluru, Karnataka, India; h HCG Bangalore Institute of Oncology, Bengaluru, Karnataka, India

**Keywords:** wide-field diffuse reflectance imaging, multispectral bimodal imaging, oral potentially malignant lesions, oxygenated hemoglobin absorption mapping, biopsy guidance, oral cancer screening and early detection

## Abstract

**Significance:** Screening and early detection of oral potentially malignant lesions (OPMLs) are of great significance in reducing the mortality rates associated with head and neck malignancies. Intra-oral multispectral optical imaging of tissues in conjunction with cloud-based machine learning (CBML) can be used to detect oral precancers at the point-of-care (POC) and guide the clinician to the most malignant site for biopsy.

**Aim:** Develop a bimodal multispectral imaging system (BMIS) combining tissue autofluorescence and diffuse reflectance (DR) for mapping changes in oxygenated hemoglobin (${\mathrm{HbO}}_{2}$) absorption in the oral mucosa, quantifying tissue abnormalities, and guiding biopsies.

**Approach:** The hand-held widefield BMIS consisting of LEDs emitting at 405, 545, 575, and 610 nm, 5MPx monochrome camera, and proprietary Windows-based software was developed for image capture, processing, and analytics. The DR image ratio (R610/R545) was compared with pathologic classification to develop a CBML algorithm for real-time assessment of tissue status at the POC.

**Results:** Sensitivity of 97.5% and specificity of 92.5% were achieved for discrimination of OPML from patient normal in 40 sites, whereas 82% sensitivity and 96.6% specificity were obtained for discrimination of abnormal (OPML + SCC) in 89 sites. Site-specific algorithms derived for buccal mucosa (27 sites) showed improved sensitivity and specificity of 96.3% for discrimination of OPML from normal.

**Conclusions:** Assessment of oral cancer risk is possible by mapping of ${\mathrm{HbO}}_{2}$ absorption in tissues, and the BMIS system developed appears to be suitable for biopsy guidance and early detection of oral cancers.

## 1 Introduction

According to GLOBOSCAN 2018 reports, 354,864 new cases of oral cancer and 177,384 deaths have occurred worldwide in 2018,^1^ and a fifth of this burden is from India.^2^ The five-year survival rate for oral cancers is around 50%, regardless of the improvement in diagnostic modalities and treatment outcomes.^3^ Early detection of pre-malignant lesions in the oral cavity is the best way to improve the quality of life of patients, to effectively manage the disease, and to improve treatment outcomes.^4^ In oral cancer diagnostics, detection of oral potentially malignant lesions (OPMLs) is of great significance. OPML such as leukoplakia, erythroplakia, and oral submucous fibrosis (OSMF) have a risk for malignant transformation of 15% to 39%, 51%, and 7% to 26%, respectively.^5^ Recent guidelines recommend an initial biopsy to assess the disease status of OPMLs.^6^ Tissue biopsy is known to be the diagnostic gold standard for OPML identification and analysis.^7^^,^^8^

In low-resource settings, conventional oral examination (COE) with white light is the standard procedure for evaluating OPMLs.^6^ The major limitation associated with COE is that it is subjective; the screening results depend on the expertise of the clinician examining the patient. Often, malignant tissue changes are discarded by COE,^9^ and dysplastic tissues can still be located within healthy oral mucosa.^10^ Evidence shows that COE is not a good discriminator of oral mucosal lesions.^11^ Clinical examination has limited value in detecting the malignant potential of OPMLs since their macroscopic appearance often does not reflect their histopathologic and molecular features; despite this, evaluation of OPMLs is still largely based on simple mucosal inspection.^12^ It is a challenging task even for experienced clinicians to locate the most malignant site for biopsy, especially in large OPMLs. This anomaly often leads to multiple or unwanted biopsies, delayed- and under-diagnosis, and patient trauma.^13^ Because tissue biopsies are intrusive, labor-intensive, and often take a few days to process and interpret, many OPMLs are not biopsied, particularly in low-resource settings.^14^ The existing adjunctive techniques, including vital tissue staining, brush biopsy, chemiluminescence, and autofluorescence imaging, do not provide sufficient diagnostic accuracy for the detection of premalignant changes in the oral mucosa.^13^^,^^15^

Tissue progression from healthy to malignant states is followed by several biochemical, morphological, and structural changes associated with the disease.^16^ These changes mirror the optical signatures derived from the interaction of light with tissues. Autofluorescence emanating from coenzymes, such as nicotinamide adenine dinucleotide (NAD), flavin adenine dinucleotide (FAD), and protoporphyrin IX (PpIX), present information on cellular metabolic activity. In contrast, reflectance spectroscopy can be used as biomarkers of changes in the tissue oxygenation levels and neovascularization.^17^ A screening device should ideally have the ability to detect early tissue transformations toward malignancy, enabling better patient care and improved survival rates.

Several clinical studies have been performed by our group^18^^–^^21^ and other researchers^22^^–^^26^ on the application of optical techniques for the detection of oral premalignant lesions. These studies have shown that non-invasive clinical adjuncts based on tissue fluorescence and diffuse reflectance spectroscopy have great potential as screening tools for the detection of oral malignancies. Our approach utilized the changes in the intensity of oxygenated hemoglobin absorption peaks at 545 and 575 nm, which were noticed in the diffusely reflected white light for classifying oral malignancies. Initial studies were carried out *ex vivo*, on surgically excised tissues of oral cavity,^18^ and were later validated through an *in vivo* clinical study.^19^ We recorded the diffusely reflected white light spectra from the oral mucosa with a fiber-optic probe and noticed that the diffuse reflectance (DR) intensity ratio (R545/R575) was lowest in normal or healthy mucosa and gradually increased to higher and higher values with increasing grades of oral cancer. The heme production is low in cancer cells owing to the reduced activity of ferrochelatase enzyme in the heme cycle, which leads to a build-up of PpIX in the cancer cells.^19^ By recording the 405-nm laser-induced fluorescence spectra of oral mucosa with an optical fiber probe, we were able to detect this increase in PpIX *in vivo* and discriminate different grades of oral cancer using a spectral ratio reference standard.^20^ Later, a widefield imaging configuration was developed with white light illumination and an external electron multiplying CCD camera to capture DR images of oral lesions at 545 and 575 nm, and the image ratio R545/R575 was utilized to screen and detect oral cancers.^21^

Most of the commercially available devices in the market, such as VELscope^®^ (LED Dental, White Rock, British Columbia, Canada) (20 to 22), Bio/Screen^®^ (Addent, CT), ViziLite PRO^®^ (DenMat, Lompoc, California), and OralID^®^ (Forward Science), rely on visualization of tissue fluorescence on excitation with violet/blue light from outside of the oral cavity, whereas Identafi^®^ (StarDental - DentalEZ, Lancaster, Pennsylvania) is an intraoral device that uses both tissue fluorescence and reflectance of tissues for oral cancer screening.^27^^–^^29^ However, the main limitation with these devices is that they are subjective, relying mostly on visual impressions; however, VELscope, Bio/Screen, and Vizilite PRO^®^ do have options for external camera attachment.^30^ Cancerous lesions often show loss of fluorescence and appear as dark areas in these images, making it impractical for a biopsy guidance application. The detection accuracies reported by these devices are poor as inflammatory tissues also show loss of fluorescence, and unwanted biopsies are a major concern. Therefore, these devices did not gain acceptance even in countries such as India where the prevalence for oral cancer is high. Although, Identafi^®^ is an intraoral bimodal device, it also is subjective and does not incorporate a camera for image capture.^31^^,^^32^ Furthermore, the use of an expensive disposable mirror adds to the cost of screening and makes it unsuited for population-based screening programs.

These limitations motivated us to develop point-of-care (POC) solutions that provide quantitative information on tissue status at the POC in real-time. In this paper, we present a hand-held multispectral wide-field imaging intraoral camera for recording of tissue autofluorescence and DR that is illumined with multiple LEDs. The monochrome USB camera is controlled through proprietary software installed on a personal computer. The captured images are processed and analyzed using a cloud-based machine-learning (ML) algorithm for real-time user feedback. With the help of pseudo-color maps representing variations in oxygenated hemoglobin absorption in tissue, we detect tissue abnormalities and locate the most malignant site in a lesion for biopsy. We also present a representative pilot study results to demonstrate the potential of the device for screening and early detection of oral cancers and guided biopsies.

## 2 Materials and Methods

### 2.1 Instrumentation

**Fig. 1:**
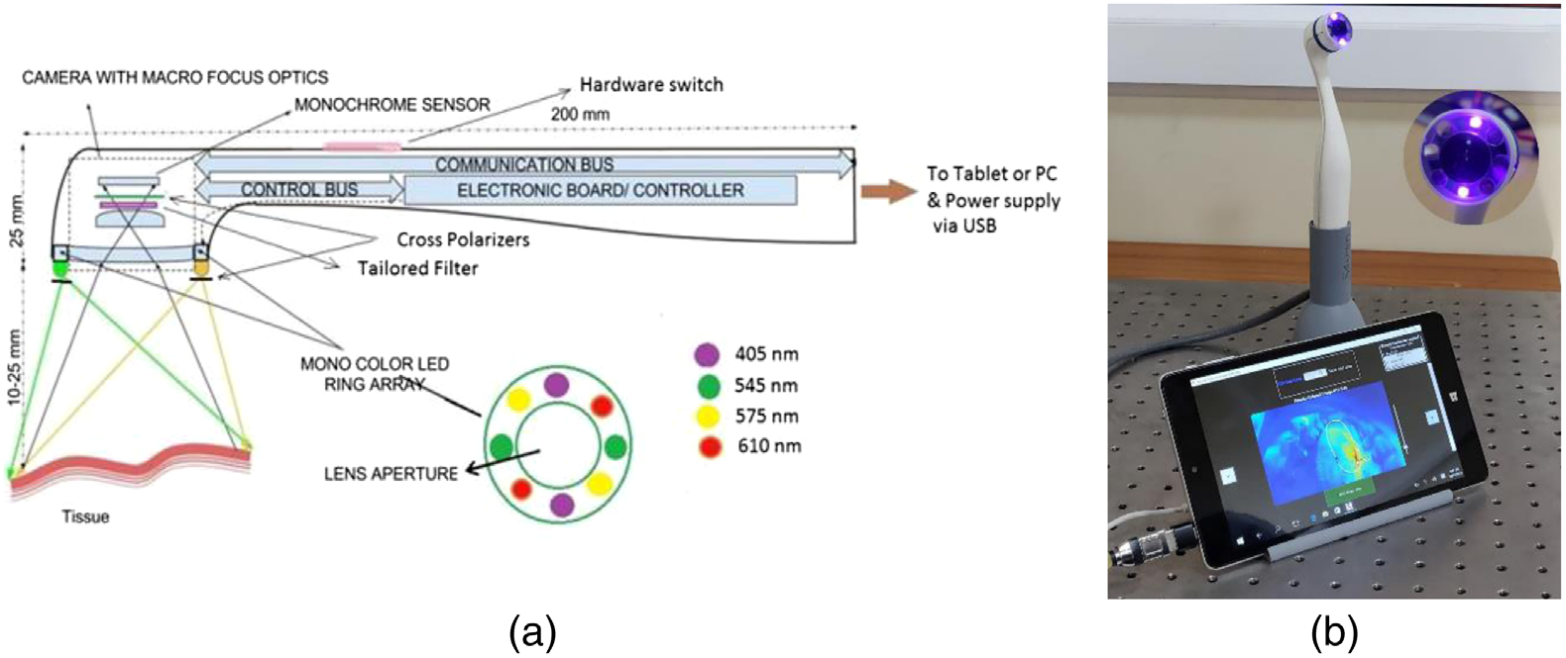
(a) Schematic of the BIMS system developed for oral cancer screening and (b) prototype of the BMIS connected to an 8 in. Windows tablet. The inset shows the probe head with narrowband LED filters mounted on the outer window.

A hand-held bimodal multispectral imaging system (BMIS) has been developed [Figs. 1(a) and 1(b)] for wide-field intra-oral screening.^33^ The BMIS consists of a sub-miniature monochrome camera (Ximea, GmbH, Model: MU9PM-MH) featuring a 5 MPx CMOS sensor (Aptina MT9P031) with 2.2-micron pixels and 2592×1944 resolution for image capture (Fig. 1). Light-emitting diodes (LEDs) situated around the camera lens, emitting at violet (405 nm), green (545 nm), yellow (575 nm), and red (610 nm) wavelengths of light, were used for tissue illumination. Narrowband interference filters (5-mm diameter, FWHM width of 8 nm) centered around 542, 577, and 610 nm were placed over a window covering the respective LEDs to ensure that their light output matches with the absorption dips of ${\mathrm{HbO}}_{2}$ at 542 and 577 nm and that the LEDs spectral outputs do not overlap. The 610-nm LED acts as a reference standard, in which ${\mathrm{HbO}}_{2}$ absorption is minimal. The light collection optics included a tailored filter that transmits tissue autofluorescence and the elastically backscattered light at 542 and 577 nm into the sensor, while blocking the 405-nm light from entering the detection system. The patented optical engine also consisted of crossed polarizers in the light illumination and collection paths to prevent specular reflections from the tissue surface reaching the sensor. The camera is connected to the USB port of a tablet or laptop with a 64-bit Windows 10 operating system [Fig. 1(b)]. The BMIS is thus configured to capture multimodal images of oral mucosa using its integrated hardware and proprietary software. More details on the BIMS imaging system regarding optical configuration, hardware, and software integration are described elsewhere.^33^

### 2.2 Tissue Classification Algorithm

In the present study, we implemented a ratio-metric algorithm (R545/R610) based on the diffusely reflected light intensities of 545 and 610 nm. This was achieved by pixel-by-pixel division of the monochrome images captured by the camera following illumination with 545- and 610-nm wavelength LEDs. The DR intensity of tissues (R) is determined by the concentration of ${\mathrm{HbO}}_{2}$ and Hb in tissues and their effective attenuation coefficients (a1 and a2) at these wavelengths using the relation:^34^

$\ln(R)=(a1*[{\mathrm{HbO}}_{2}]+a2*[\mathrm{Hb}])+\ln(k)$, where k is a constant term, which can be removed by calibration, and total hemoglobin concentration (tHb) and oxygen saturation ($S_{t}O_{2}$) are defined as:

$$\mathrm{tHb}=[{\mathrm{HbO}}_{2}]+[\mathrm{Hb}],\;\text{and}\;S_{t}O_{2}=[{\mathrm{HbO}}_{2}]/t[\mathrm{Hb}].$$

The DR intensity at any two wavelengths can be used to calculate the concentration of ${\mathrm{HbO}}_{2}$ and Hb so that the total concentration of hemoglobin and oxygenation can be determined. The wavelength 545 nm belongs to one of the isosbestic points of Hb, where ${\mathrm{HbO}}_{2}$ and Hb have strong absorption. At this wavelength, the reflectance intensity is more sensitive to changes in ${\mathrm{HbO}}_{2}$ than at other wavelengths in the spectrum. In the 610- to 630-nm wavelength range, the absorption coefficient of Hb is 7 to 8 times higher than that of ${\mathrm{HbO}}_{2}$ so that absorption at this wavelength is primarily due to Hb present in the tissue (Fig. S1 in the Supplementary Material). Therefore, the extent of malignant transformation can be assessed from the increase in the R610/R545 ratio in the region of interest (ROI) marked surrounding the lesion, with reference to the tissue fluorescence image (F405).

It is known that reflectance and light scattering spectroscopy provide information on morphologic and structural changes in tissue architecture and epithelial cell nuclei and polarized light reflectance spectroscopy provides quantitative information on tissue morphology that could be used for non-invasive and real-time detection of epithelial neoplasia.^35^ The tissues of various anatomical sites of the oral cavity have differing optical and spectral characteristics. Therefore, we grouped the tissues of different anatomical sites into keratinized and non-keratinized types, with the former consisting of gingiva, vermillion border of the lip, dorsal tongue, hard palette, and lower/upper alveolus and the latter consisting of left/right buccal mucosa, lateral/ventral tongue, floor of the mouth, inner lip, and gingivo-lingual/buccal sulcus. Therefore, two sets of R610/R545 ratio algorithms were developed to provide user feedback during the screening process, based on the site of the lesion. When biopsies are taken from the sites identified as most malignant, the R610/R545 value associated with the biopsy site is uploaded to the cloud along with the pathology report. The new ratio values and the corresponding diagnostic results are merged into the ML algorithm, making it robust over a period of time. When more and more data sets are incorporated into the algorithm and site-specific algorithms become available, the accuracy of screening and disease prediction improves.

### 2.3 Clinical Trials

The BMIS was validated through a multicentric clinical study covering six hospitals (HCG Hospital, JSS Dental College, SUM Hospital, Govt Dental College Kottayam, Sri Balaji Dental Hospital, and Dayanand Sagar Dental College). Individual ethical approvals were obtained from the respective ethics committees of these hospitals, and the trial was registered prospectively at the Clinical Trial Registry of India with Ref No. CTRI/2017/10/010125 dated October 18, 2017. The study details were explained in detail to the patients who participated in the study, and written informed consent was obtained before initiation of any study-related procedures.

**Fig. 2:**
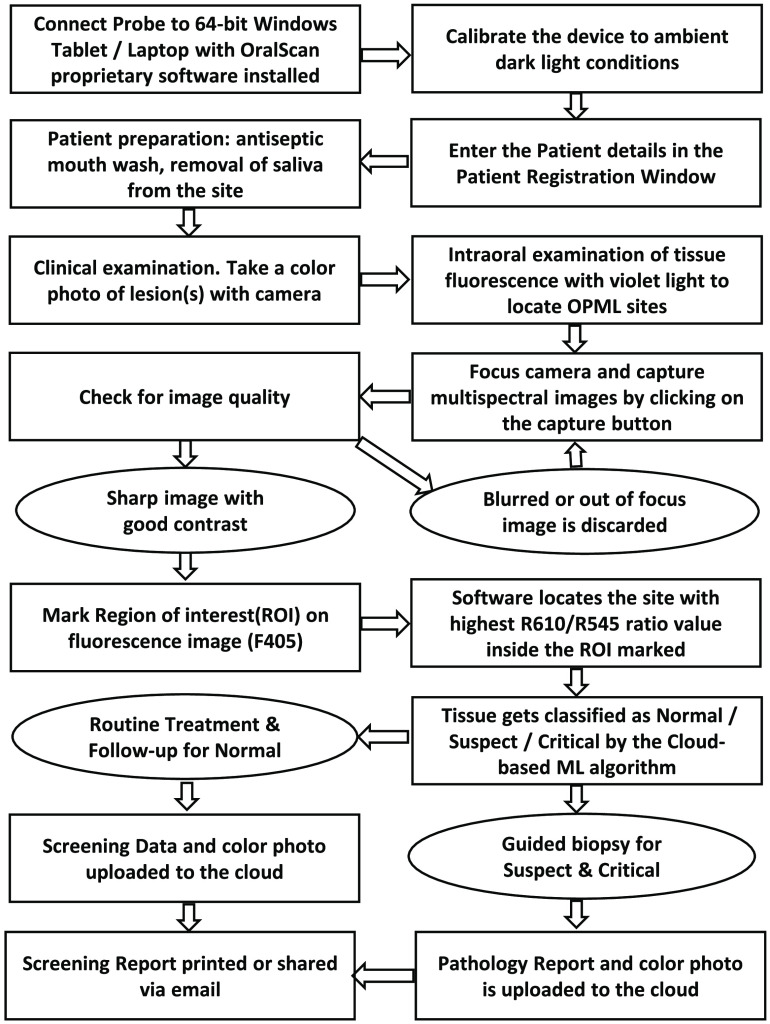
Schematic diagram showing the working process of the BMIS.

All clinical measurements using the BIMS probe were carried out in a dark room following the calibration procedure given in Sec. 2.5. Figure 2 shows a schematic diagram of the steps involved in the working process of the instrument. The entire screening process takes no more than 5 mins to complete for a patient. The immediate display of the captured images on the computer screen helps with image quality checks and recapture when necessary. Once the ROIs are marked with reference to the tissue autofluorescence image, the software program locates the pixel with the maximum ratio value in the ROI and assigns the mean value of a 5×5 matrix surrounding that pixel as the DR ratio (R610/R545) value for tissue classification as normal, suspect, or critical based on feedback from the cloud-based ML algorithm. A guided biopsy shall be taken if required. The graphical user interface (GUI) and work flow are organized to make the screening process intuitive and straightforward. The screening data is saved in the local drive of the computer and uploaded to the cloud for backup and algorithm refinement. The storage and integration of patient data from different screening centers on a single cloud platform facilitate uniformity in analytics and development of a robust ML algorithm for the benefit of all users.

### 2.4 General Description of the Data Set

A total of 336 patients were recruited for this study, out of which 89 patients (118 sites) underwent biopsy procedures. Multiple sites of the same patient showing different grades of pathology were included as a separate dataset. Images from 118 intraoral sites were further scrutinized; four data were excluded due to lack of histopathologic report, six data were removed due to poor image quality (blurring), and 10 data were excluded due to non-uniformity in light illumination. Finally, 89 sites from 65 patients were included in the study. The average age (±SD) of the patients was 51±(14) years. The inclusion criteria incorporated selection of patients presenting with OPML, such as leukoplakia, erythroplakia, OSMF, dysplasia, and moderate to well-differentiated squamous cell carcinoma (SCC) in the case of malignant tissues. The exclusion criteria encompassed patients who have undergone prior cancer treatments, have systemic conditions that contraindicate biopsy, have used any oral medication for at least four weeks, or were not willing to participate in the study. OSMF cases are mostly diagnosed from their clinical characteristics; hence they are not biopsied as a surgical intervention may induce further disease progression. Various types of pathological specimens screened using the probe are given in Table 1.

**Table 1:** Clinical status of different pathological specimens included in the study.

Tissue type	# of patients
**OPML**	
Carcinoma *in situ*	1
Lichen planus	2
Fibro epithelial polyp	1
Leukoplakia	27
Hyperplasia	8
Ulcer	1
**Malignant lesions**	
Poorly differentiated SCC	7
Moderately differentiated SCC	14
Well differentiated SCC	28

### 2.5 Calibration of the Instrument

The BMIS is calibrated to the ambient (dark/dim) light conditions by positioning the probe head over the calibration unit that houses a tissue phantom, maintaining a working distance of 2 cm with the probe tip. The calibration process is initiated by clicking on the calibration/settings button on the software window. The display shows calibration successful on completion of the procedure that involves switching on/off of the different LEDs and varying the exposure settings of the camera for each set of LEDs such that all four images captured by the camera are optimally exposed during the recording process. This is achieved by limiting the mean pixel intensity of the four different frames to within 2.5% to 4% of their mean pixel value based on the number of retries done to successfully calibrate the device. Once calibrated, there is no need to recalibrate the instrument unless the room lighting conditions change.

### 2.6 Image Acquisition and Analysis of the Tissue Characteristics

**Fig. 3:**
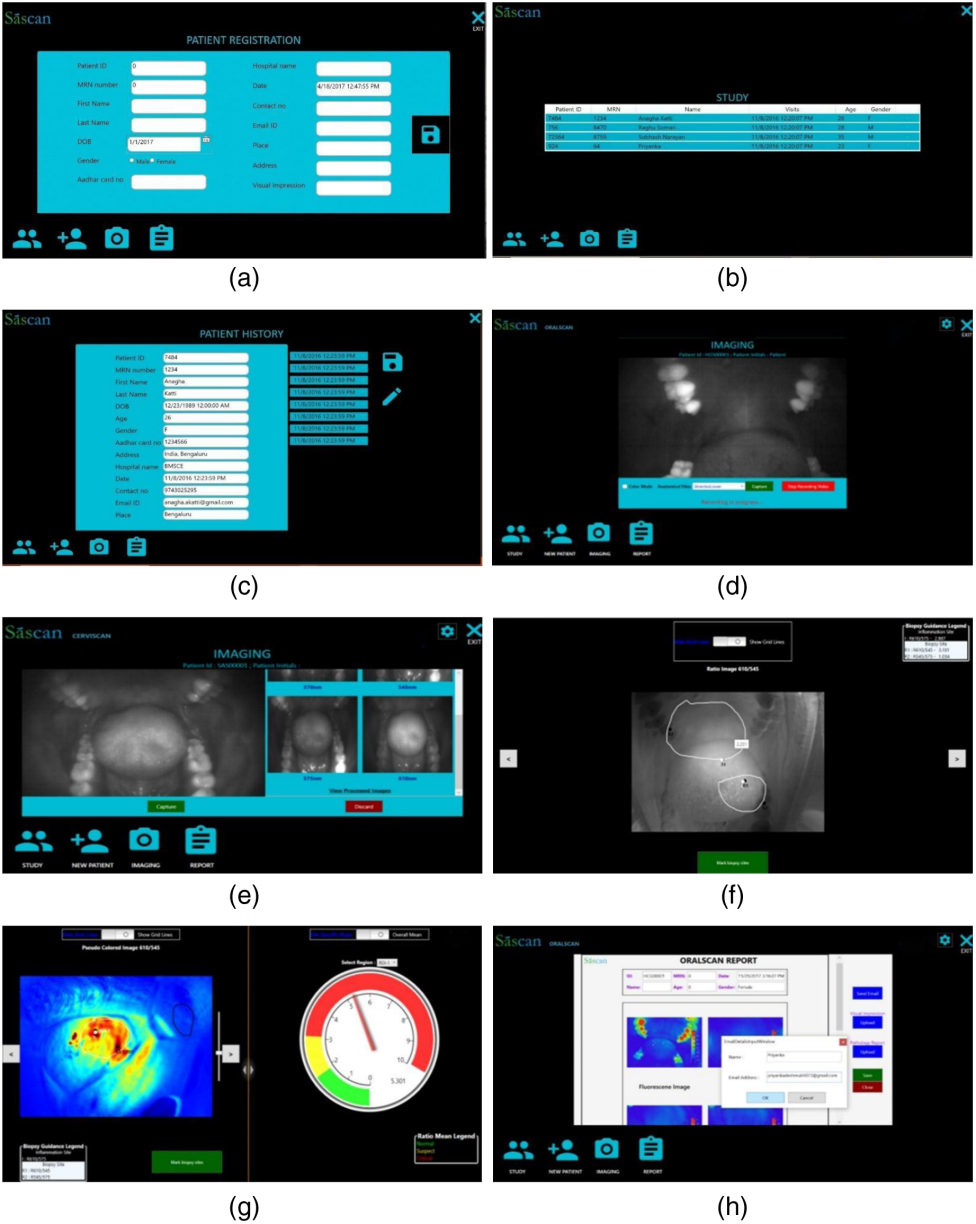
Different modules of BMIS software for image capture and analysis. (a) Opening window for patient registration; (b) patient study window to select patient for screening; (c) patient history window for viewing/editing of captured images; (d) video recording window; (e) image capture and display window; (f) window for ROI marking of patient normal and abnormal areas; (g) PCM of R610/R545 image ratio for biopsy guidance, and the CDD for tissue status assessment, with green representing normal, yellow representing OPML (Suspect), and red representing SCC (Critical); and (h) report page with features for biopsy result entry, data upload to the cloud server, and report sharing.

During patient enrollment, in addition to patient name and contact details, mandatory information related to age, habits, and visual/clinical impressions are collected, whereas the medical record number is autogenerated [Fig. 3(a)] in the patient information section of the software program. The patients are seated comfortably on a dental chair and are examined following the sequence shown in Fig. 2. An oral rinse with water followed by saline wash or antibacterial mouthwash is recommended. The lesion photo is captured on a mobile phone camera for reference. The screening probe is now wiped cleaned with isopropyl alcohol and covered with a thin and transparent plastic cling wrap film (Polyvinyl Films, Inc, Massachusetts, Model: Kirkland stretch-tite or equivalent food wrap film) to maintain hygiene and prevent probe contact with the oral cavity. The room light is set to the ambient dark conditions used for calibration. On powering the probe, the violet LED is switched on. This light is used to observe tissue abnormalities, locate OPMLs by tissue autofluorescence, and identify areas for detailed examination.

The software has provisions for marking the suspicious sites for detailed investigation in an anatomical diagram based on visual observation. On selecting the imaging mode, a live view of the oral cavity is possible in a new window [Fig. 3(d)] in monochrome (grayscale) or pseudo color to locate the lesion spread and visually assess the extent of tissue health. The software has provisions for video and image capture. During screening, care should be taken to minimize specular reflection from oral mucosa by keeping the mucosal surface dry with cotton swab and holding the probe at an angle to the imaging surface. Once the OPML is located, the camera is focused by varying the distance between the probe and the tissue, and four monochrome images are captured sequentially by a single press of the capture button on the device or the software window. The quality of the images displayed on the screen [Fig. 3(e)] is checked to ensure that all four images are sharp without motion artifacts due to patient or probe movement and are free of any bright spots due to reflection from saliva, explorer probe mirror, or illumination non-uniformity. Although the image capture takes <90  s, probe movement can be minimized by resting the hand holding the probe on the patient’s body or some other stationary object. If the images are not focused or intensity saturation is noticed in any region, the captured images can be discarded and a fresh set of images recaptured.

The four images captured sequentially and illuminated with four different LEDs emitting at 405, 545, 575, and 610 nm are designed at F405, R545, R575, and R610. All four monochrome images are displayed on screen along with the R610/R545, R610/R575, and R545/R575 ratio images in monochrome/pseudo color [Fig. 3(e)]. The outer border of the OPML or malignant lesion is marked as the ROI using the mouse pointer or stylus pen on the touch screen based on the extent of the pseudo color map (PCM) of tissue fluorescence. In addition, an apparently healthy region that is free of tissue inflammation is marked on the same anatomical region adjoining the lesion with a low R610/R575 ratio value^36^ [Fig. 3(f)]. The software program automatically determines the mean DR image ratio (R610/R545 and R610/R575) in the ROIs of healthy mucosa and the highest ratio value in the ROIs of R610/R545, R545/R575, and R610/R575 in the OPML. The DR ratio values along with the processed and captured images are now pseudo color mapped to visualize variations in the DR ratio across the lesion. The extent of color coding can be altered by up or down movements of the cursor on the PCM adjuster located beside the image display window. The screening result is presented in a color-coded display diagram (CDD), with the pointer showing the highest R610/R545 ratio value in the ROI, representing the most malignant site in the OPML. Green color in the CDD represents healthy/normal tissue, yellow represents suspect (OPML), and red represents critical (malignant) lesions.

Based on the variance of the R610/R545 ratio value representing the most malignant site in the ROI with respect to the mean ratio value of an adjoining healthy region, it would be possible for the clinician to decide on whether a biopsy is required or habit cessation and follow-up would suffice. On completion of the screening process, the value of DR ratios, such as R610/R545, R545/R575, and R610/R575, are populated in an excel file, and the captured images and data files are pushed to the cloud automatically on internet connectivity. Later, when the ROI is remapped or the pathology reports and lesion photos are uploaded, the cloud data also are refreshed automatically.

## 3 Results

**Fig. 4:**
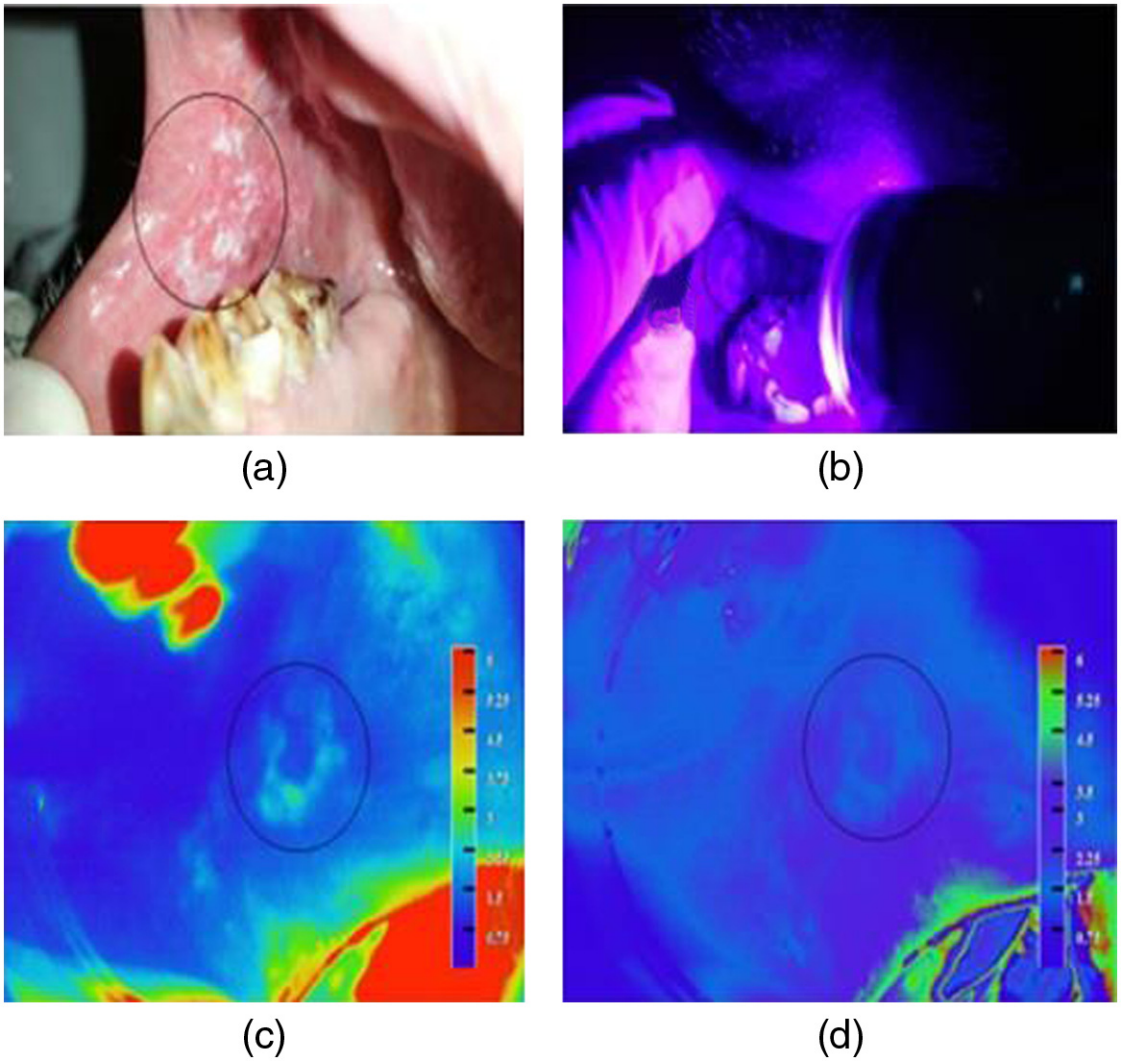
Homogenous leukoplakia on the right buccal mucosa: (a) photo of the lesion, (b) live view with violet light, (c) PCM of tissue fluorescence, and (d) PCM of R610/R545 image ratio.

**Fig. 5:**
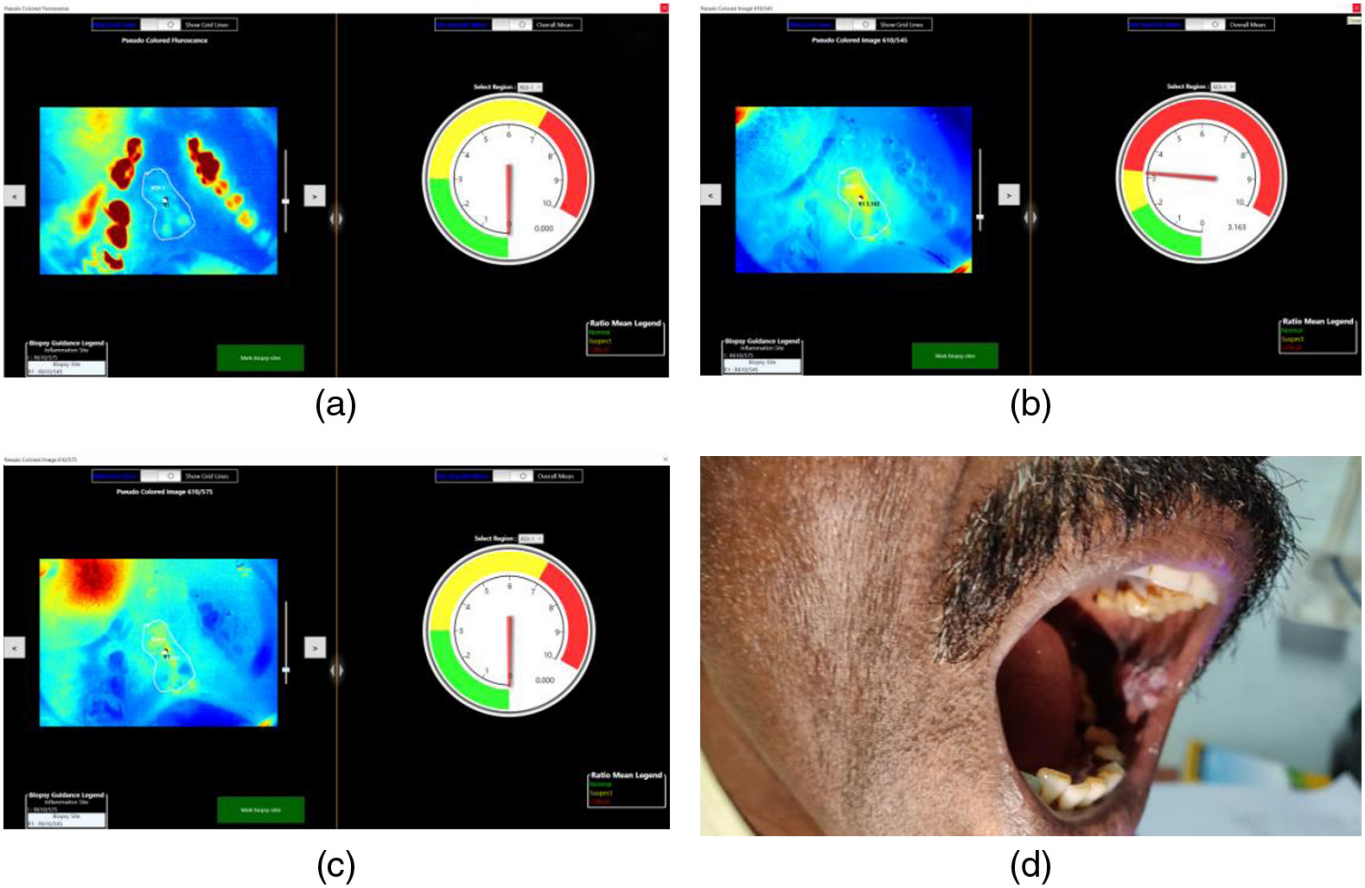
Screening results of a 59-year-old patient with speckled leukoplakia on the left buccal mucosa: (a) PCM of tissue fluorescence, (b) PCM of R610/R545 image ratio with the tissue status displayed on the CDD, (c) image ration R610/R575 representing tissue inflammation, and (d) lesion photo. The lesion border is marked on the screen based on visual impression and PCM of tissue fluorescence. The software program locates the site with the highest ratio value, inside the white ROI marked on the PCM of R610/R545 ratio, as the most malignant site for biopsy.

Fluorescence visualization of homogenous leukoplakia in the right buccal mucosa of a patient with violet light illumination is depicted along with the PCM of the corresponding autofluorescence and the DR image ratio (R610/R545) in Figs. 4(a)–4(d). The PCM of tissue fluorescence and DR ratio maps can be visually enhanced as per user perspective to locate the lesion spread. Figures 5(a)–5(d) show the PCM images tissue fluorescence, the DR image ratio R610/R545 representing malignant transformations, R610/R575 representing tissue inflammation, and the photo of the oral cavity of the patient with speckled leukoplakia on the left buccal mucosa. The ROI of the lesion is marked on the fluorescence image outlining the lesion, while the ROI representing patient normal is marked in an adjoining area belonging to the same anatomical site, carefully avoiding areas of tissue inflammation with high R610/R575 values. Once the ROI is marked, the user can visualize the most malignant site in the lesion for biopsy from the PCM of the R610/R545 ratio that represents the absorption changes due to ${\mathrm{HbO}}_{2}$ in tissue. The corresponding ratio value with respect to the most malignant site also is displayed on the screen along with the mean value of the DR image ratio from the adjoining site marked as patient healthy. As the mouse pointer has a ratio drop-down display feature, the DR ratio values across the lesion can be explored by moving the mouse cursor across the lesion to review and locate if any malignant sites of the oral cavity went unnoticed during the ROI marking process.

### 3.1 Machine Learning Algorithm Development

**Fig. 6:**
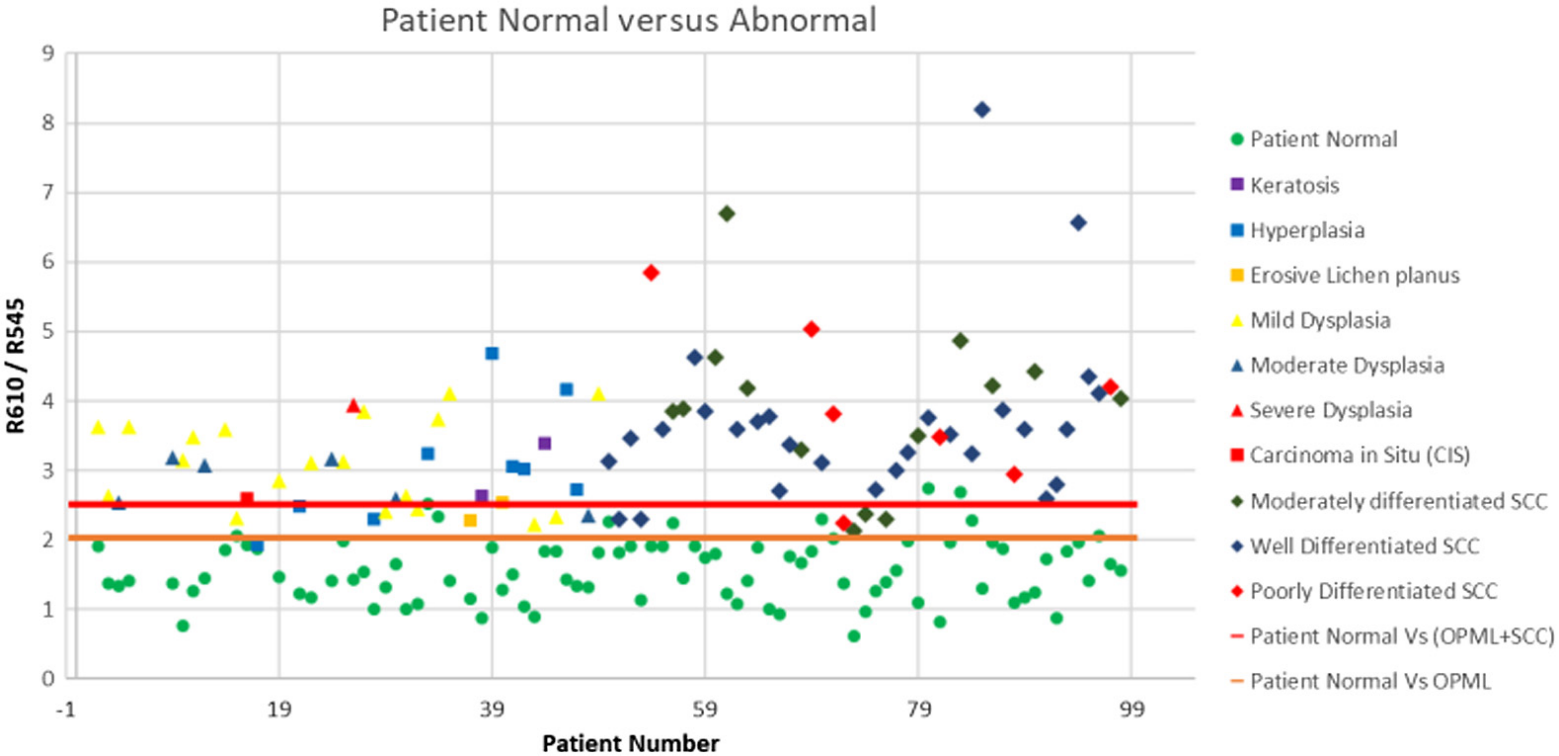
Scatter plot ratio (R545/R610) algorithm for classifying abnormal and OPML tissues of the oral cavity from patient normal tissues, with the red discrimination line drawn at the mean of (OMPL + SCC) ratio values and the patient normal ratio values and the orange line drawn at the mean of OPML and patient normal ratio values.

To classify the various tissue types involved in the study, we utilized the ratio (R610/R545) of the diffusely reflected light from squamous epithelium at 610 nm (R610) and 545 nm (R545). Figure 6 shows scatter plot diagrams representing the DR image ratio (R610/R545) versus patient number for discriminating potentially malignant (OPMLs) and abnormal tissues involving OPMLs and SCC, from apparently healthy tissues in patients. The tissues included in the algorithm were of varied morphology and structure, covering all anatomical locations in the oral cavity (Table S1 in the Supplementary Material). The DR ratio values of healthy tissues relate to the mean R610/R545 ratio of the adjoining healthy region marked in the patient mucosa belonging to the same anatomical site, with no or minimal signs of tissue inflammation.^36^

The mean DR intensity ratio (R610/R545) for healthy samples was found to be 1.56, whereas for OPML sites it was 3.03 and for SCC tissues the value was higher at 3.44. This shows that the DR image intensity ratio (R610/R545) increases with the increase in malignancy, with the lowest values for normal/healthy mucosa and increasing to higher and higher values as the tissue transformation takes place from OPML to SCC. The discrimination threshold lines shown in Fig. 6 were drawn at the mean values of the data sets involved. For instance, the discrimination threshold line between healthy tissues and OPML was calculated and drawn at the mean of the average ratio values of patient healthy and OPML data sets. In Fig. 6, the discrimination threshold lines were drawn at 2.028 and 2.501, respectively, for classifying OPML and abnormal (OPML+SCC) tissues from patient normal tissues covering all anatomical sites of patients that underwent a guided biopsy procedure.

### 3.2 Diagnostic Accuracy

Table 2 represents the diagnostic ability of the device obtained with the DR ratio (R610/R545) algorithm for discriminating OPML and abnormal tissues (OPML + SCC) from all anatomical sites with adjoining normal tissues from the same anatomical site. Table 2 also presents the improvement in diagnostic accuracy when healthy volunteer data are used instead of patient normal tissues in the R610/R545 ratio algorithm for buccal mucosal malignancies. To evaluate the diagnostic accuracy, we implemented the discrimination threshold as a cutoff value for differentiating healthy tissues from the different tissue pathologies involved. The algorithm shown in Fig. 6 for discriminating OPML from patient normal tissues with a cut-off value at 2.03 resulted in a sensitivity of 97.5% and specificity of 92.5% with positive predictive value (PPV) and negative predictive value (NPV) values of 0.93 and 0.97, respectively, whereas for discrimination of patient normal tissues from abnormal tissues, a sensitivity of 82% and specificity of 96.6% were obtained with the cutoff value at 2.5 and PPV and NPV values of 0.96 and 0.84, respectively.

**Table 2:** Diagnostic ability of R610/R545 ratio for differentiating patient normal tissues from OPML lesions and abnormal tissues (OPML and SCC).

Diagnostic parameters	All anatomical sites		Buccal mucosa		
	Patient normal versus OPML	Patient normal versus abnormal (OPML + SCC)	Patient normal versus OPML	Healthy volunteer versus OPML	Healthy volunteer versus abnormal
Sample size (n)	40	89	27	27	43
Cut-off value	2.028	2.501	2.214	2.112	2.287
Sensitivity (%)	97.5	82.02	96.3	96.3	95.34
Specificity (%)	92.5	96.63	96.3	100	100
Positive predictive value (PPV)	0.929	0.961	0.963	1.00	1.00
Negative predictive value (NPV)	0.974	0.843	0.963	0.964	0.955
ROC-AUC	0.987	0.989	0.995	1.00	1.00

**Fig. 7:**
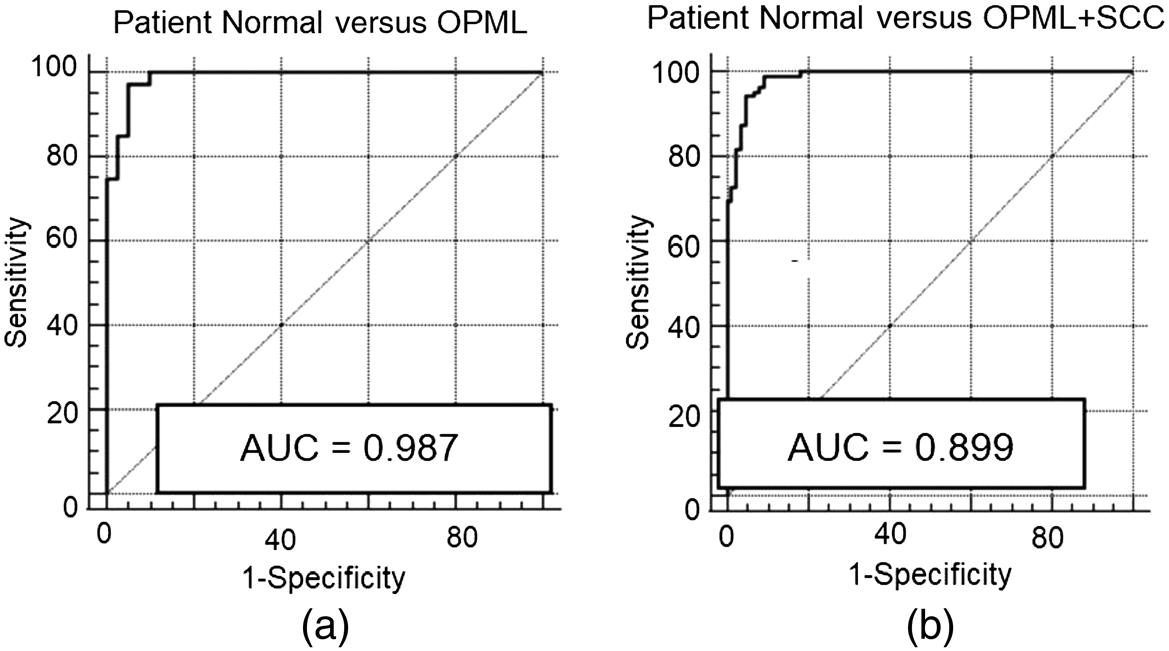
ROC-AUC using R610/R545 intensity ratios for classifying (a) patient normal versus OPML sites and (b) patient normal versus abnormal (OPML + SCC) tissues in the oral cavity.

The receiver operator characteristic-area under the curve (ROC-AUC) was also computed to evaluate the diagnostic performance of the R610/R545 ratio algorithm for tissue discrimination. The ROC-AUC values (Fig. 7) were found to be 0.987 and 0.989, respectively, for discrimination of OPML tissues and abnormal (OPML + SCC) tissues from patient normal tissues. The diagnostic accuracies given in Table 2 establish the significance of utilizing a DR ratio (R545/R610) algorithm, using the intensity of the ${\mathrm{HbO}}_{2}$ absorption peak at 545 nm to that of the non-absorbing wavelength of 610 nm, for the detection of tissue pathologies using our non-invasive bimodal imaging system implemented on a multispectral imaging platform.

### 3.3 Significance of Interquartile Range Values

The spread of DR intensity ratios in clinically healthy, potentially malignant, and malignant lesions can be explained in terms of median and interquartile range (IQR) values. In the present study, clinically healthy tissues showed a median value of 1.41 for the R610/R545 image ratio and IQR of 0.55 (0.765 to 2.524), and no outliers were identified (range: 0.452 to 2.636).

For potentially malignant, the median was found to be 2.53 and the IQR was 0.98 (1.94 to 4.69). In the case of potentially malignant tissues, there are no outliers (1.055 to 4.985). For malignant tissues, the median was 3.589 and the IQR was 1.07 (2.132 to 8.189). About 5/49 of the malignant cases fall above the maximum range (2.29 to 5), indicating that these tissues may be more aggressive or have a poor prognosis. As compared with an earlier report, the median and IQR values in the present study are slightly higher but are within the desired range.^19^

### 3.4 Site-Specific Algorithms and Effect on Diagnostic Accuracy

**Fig. 8:**
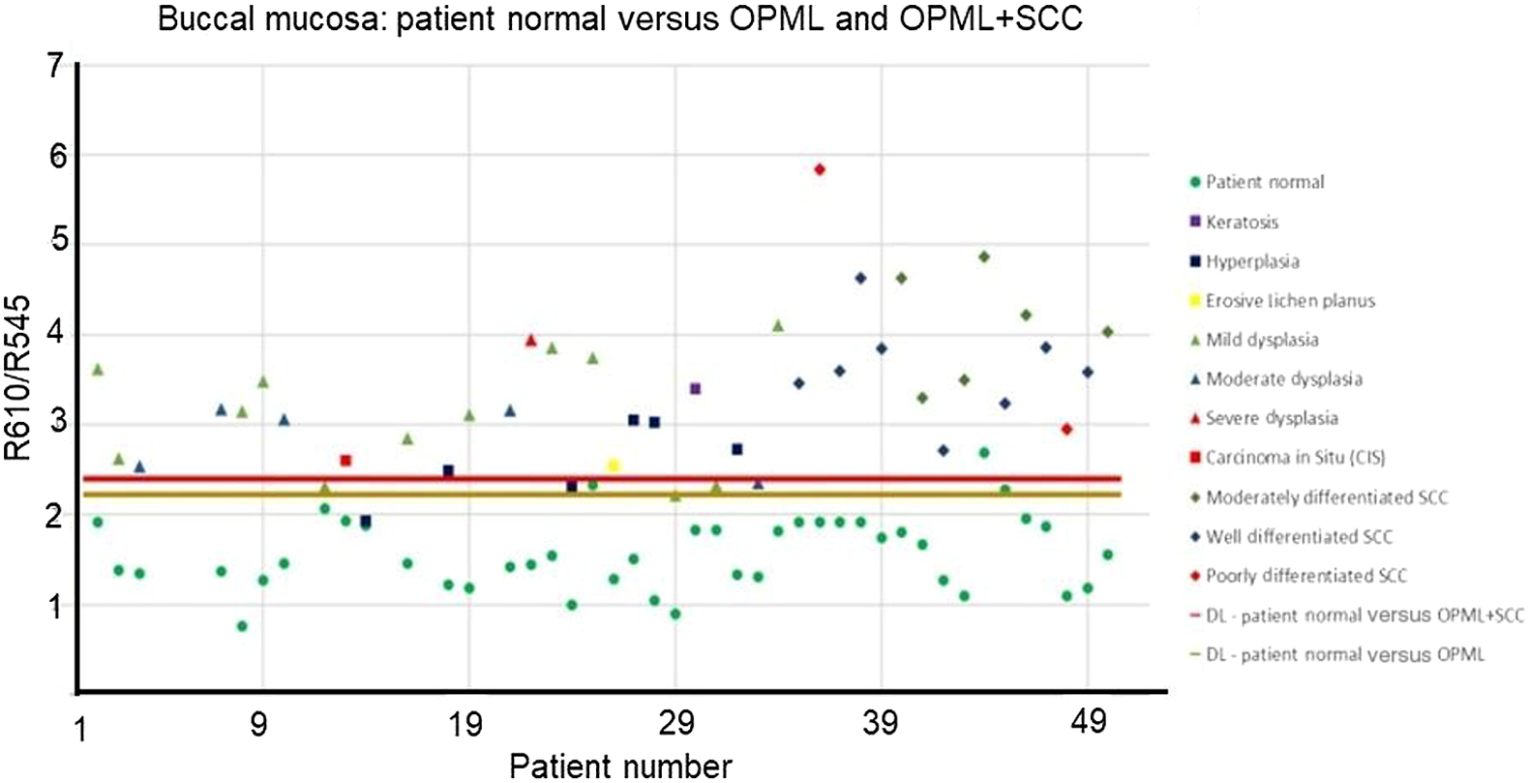
Scatter plot algorithm of R610/R545 ratio for classifying OPML (n=27) and abnormal (OPML + SCC) tissues (n=43) of the buccal mucosa with patient normal. The red line discriminates abnormal tissues from patient normal, and the brown line discriminates OPML from patient normal.

**Fig. 9:**
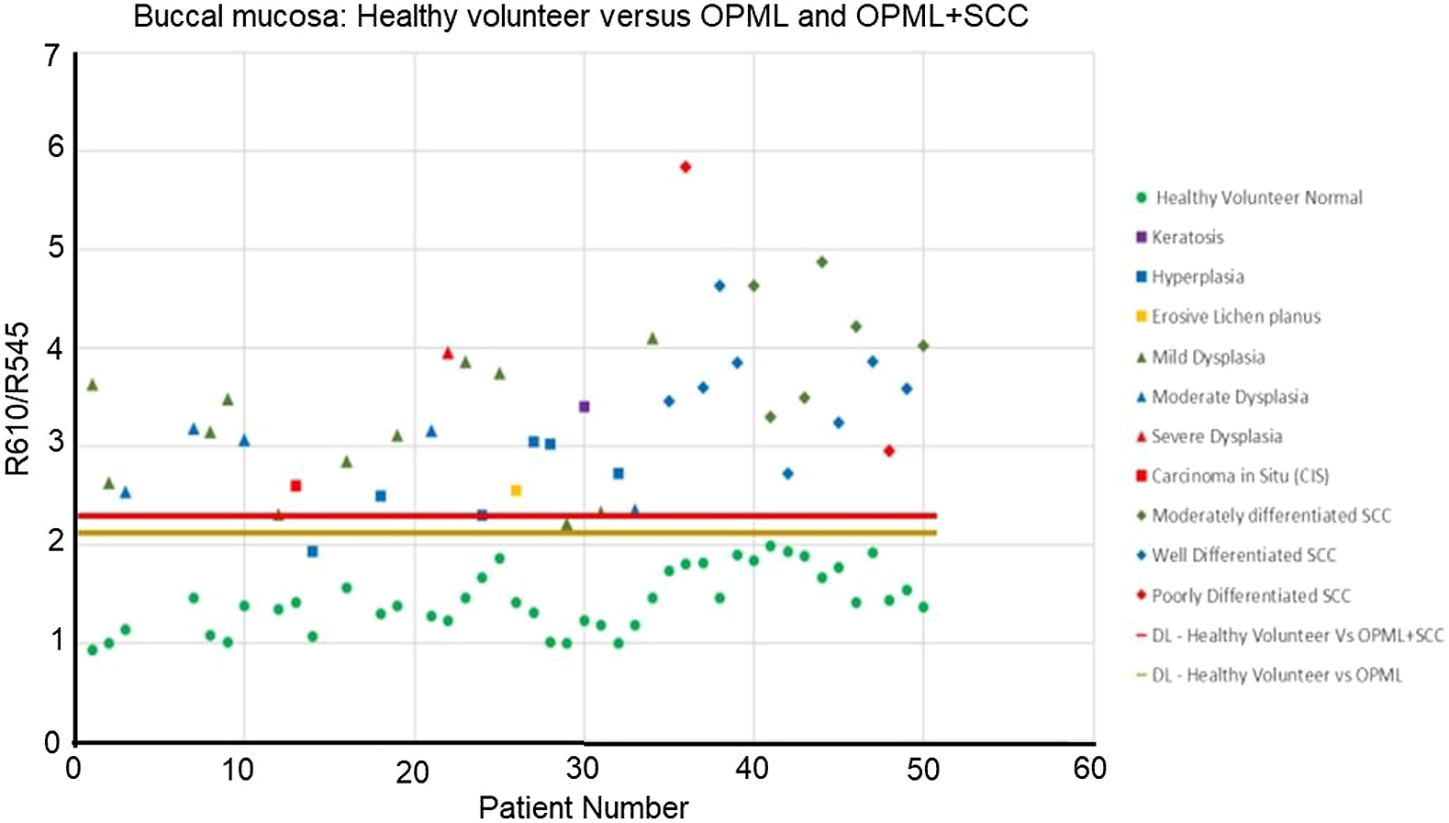
Scatter plot algorithm of R610/R545 ratio for classifying OPML (n=27) and abnormal (OPML + SCC) tissues (n=43) of the buccal mucosa with healthy volunteer data from buccal mucosa. The red line discriminates abnormal tissues from healthy normal, and the brown line discriminates OPML from healthy normal.

Previous studies have demonstrated significant differences in the spectral properties between different anatomical sites in the oral cavity.^37^^,^^38^ Improvements in the tissue classification parameters such as PPV and NPV were observed when site-specific classification was used to discriminate head and neck SCC.^39^ To test this hypothesis on existing patient data, we collated data of patients with buccal mucosal abnormalities. We also recorded the DR ratio (R610/R545) from the left and right buccal mucosa of healthy volunteers with no history of alcohol, smoking, or tobacco usage. This data set was used to replace the patient data in the site-specific (buccal mucosa) scatterplot algorithm (Fig. 8) developed for discrimination of OPML and abnormal sites from patient normal data sets. The R610/R545 algorithm developed for buccal mucosa using healthy volunteer data instead of patient normal is shown in Fig. 9.

**Fig. 10:**
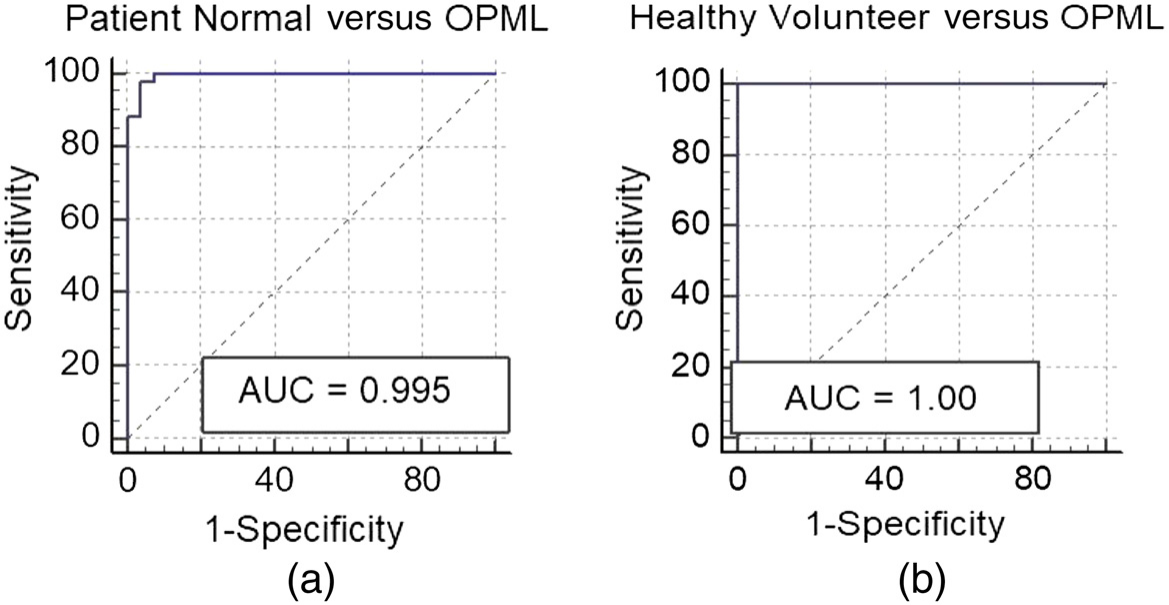
ROC-AUC plots derived from the diagnostic accuracy values obtained from the R610/R545 ratio algorithm for buccal mucosa, (a) patient healthy normal versus OPML sites and (b) healthy volunteer normal versus OPML.

Table 2 shows the number of patients in each category, the discrimination line (DL) cut-off values, sensitivity, specificity, NPV, PPV, and the corresponding ROC-AUC values achieved for both cases. The improvement in the diagnostic accuracy observed on substitution of patient normal data with healthy volunteer buccal mucosa data was primarily an improvement in the specificity value from 96.3% to 100% with a concomitant increase in the PPV from 0.96 to 1 (Table 2), which was primarily due to the lowering of the DL from 2.214 to 2.112. The corresponding ROC-AUC curves for discrimination of OPML in the buccal mucosa of these patients are plotted in Fig. 10. The ROC-AUC values increased from 0.995 to 1 when healthy volunteer data were used instead of patient normal for detection of OPML sites in the buccal mucosa.

## 4 Discussions

In recent times, there has been an increased interest in applying optical spectroscopy and imaging techniques for tissue diagnostics. These optical techniques are mostly non minimally or minimally invasive in nature, use non-ionizing radiation without any contrast agents, and have the ability to monitor patients over a period of time. The study results demonstrate the ability of using BMIS for screening and detection of OPMLs in the oral cavity. In addition, it can be used as a tool for guided biopsies by locating the most malignant site in the lesion for tissue biopsy and pathological confirmation of the grade of cancer with improved accuracy. This could potentially avoid multiple biopsies, minimize patient trauma, and reduce treatment costs. Furthermore, the BIMS can be used to enhance the ability of the surgeon to locate the tumor margins, such that complete resection of the lesion is possible by providing enough margins during surgical interventions and minimizing functional deficits. The wavelength (405 nm) used in this study to estimate tissue abnormalities from autofluorescence matches with the Soret band of PpIX, which is a precursor in the heme production cycle. Since the ferrochelatase enzyme is inhibited in cancer cells, there is an increase in PpIX fluorescence from cancer cells. However, other biochemical constituents present in tissue that absorb at this wavelength, such as FAD and NADH, also absorb this light, and their concentration changes during cancer development could alter the overall emission characteristics of OPMLs.

The green LED light at 545 nm overlaps with the prominent absorption band of oxyhemoglobin located at 542 nm. The red LED (610 nm) emission is at a non-absorbing wavelength for ${\mathrm{HbO}}_{2}$ and belongs to a region where scattering predominates and Hb has a higher absorption. Therefore, the diffusely reflected image of oral mucosa at 610 nm serves as a reference standard to map ${\mathrm{HbO}}_{2}$ absorption, as compared with using diffusely reflected light intensity at 575 nm.^18^^,^^19^^,^^34^ To confirm this, for the same set of data shown in Table 3, we plotted the scatterplot algorithm for the R545/R575 ratio and got a sensitivity of 37.5% and specificity of 86.2%, with a PPV of 0.556 and NPV of 0.75 for classification of patient normal from OPML, with a cutoff values of 1.561 (Table S2 in the Supplementary Material). In comparison, the sensitivity and specificity obtained for the R610/R545 ratio algorithm were 97.5% and 92.5%, respectively, (Table 2) for discrimination of OPML from patient normal.

**Table 3:** Showing the variation among intensity ratios using median and IQR.

Tissue characteristics	Q1	Q3	Median	IQR (Min–Max)
Healthy	1.27	1.82	1.41	0.55 (0.77–2.52)
Potentially malignant	2.53	3.51	3.04	0.98 (1.94–4.69)
Malignant	3.11	4.18	3.59	1.07 (2.13–8.19)

To test whether the high diagnostic accuracy for OPML is attributed to the large number of leukoplakia cases (14) involved in sample size (Table S1 in the Supplementary Material), we plotted the R610/R545 and R545/R575 ratio algorithms separately for leukoplakia cases involved in the study. It was seen that leukoplakia cases alone can be discriminated from normal with a sensitivity of 87.5% and specificity of 100% with the R610/R545 ratio algorithm as compared 97.5% and 87.5% for the algorithm covering all OPML cases with leukoplakia also included (Table S2 in the Supplementary Material).

Since tissue progression from normal tissues to OPML and SCC is accompanied by changes in tissue metabolism, structure, and morphology, these biochemical and morphological changes are manifested in the diffusely reflected spectral signatures derived from the tissue.^40^ We strongly believe that the wavelengths used in the present study, viz., 545 and 610 nm, correlate to the changes in tissue vasculature and scattering during cancer development.

In the present study, apparently normal tissues of patients were taken as control. Also, the region contralateral to that of the diseased region or a few centimetres away from the OPML and free of inflammation was taken as control. The primary cause for malignancies in the head and neck region include smoking, alcohol consumption, and betel-quid chewing.^41^^,^^42^ The carcinogens associated with the development of malignancies in the head and neck region are known to not only induce alterations in the tumor site but also affect the entire organ site.^43^ This phenomenon is referred to as field cancerization (FC), and the changes manifested in the tissue due to FC include changes in tissue microvasculature, alterations in the nuclear size, and density.^44^^,^^45^ One of the issues attributed to the decreased diagnostic ability is the FC effects in the oral cavity of patients. To validate this, we compared the DR ratio (R610/R545) values from the buccal mucosa of healthy volunteers for the discrimination of potentially malignant lesions and noticed that the specificity values improved from 96.3% of 100% when healthy volunteer data were used instead of patient normal for buccal mucosal tissues (Fig. 9). The results are in agreement with our previous study, in which we implemented and established algorithms for all of the sites combined and an algorithm specifically for the buccal mucosa.^19^ The diagnostic ability of the algorithm specific to buccal mucosa was steadfastly higher for discriminating benign, dysplastic, and malignant lesions. In a similar study, Hu et al.^39^ reported an increase in PPV and NPV when using tissue-specific classification algorithms, which resulted in the false-positive rates declining by 34%. The results from the present study indicate that site-specific algorithms implemented for the detection of lesions in the oral cavity increase diagnostic accuracy, which in turn reduces the number of false-negative rates. We also noticed that ulcers of the oral cavity show R610/R545 ratio values in the range of 2 to 2.6 (data not shown in Fig. 6), well below the malignant range (>3.6), which helps us to avoid unwanted biopsies from traumatic ulcers.

OSMF is characterized by oral inflammation, increase in submucosal collagen, and formation of fibrotic bands in the oral cavity that increasingly limit mouth opening. Increasing fibrosis causes blanching of oral mucosa, which results in a marble-like appearance. During the present study phase, nine OSMF cases were also screened, but they were excluded from the DR ratio algorithm and analysis as biopsies were not taken for histopathology. OSMF is a potentially malignant disorder with FC characteristics and biopsies usually taken only when clinical observations warrant it. The cases investigated had DR ratio (R610/R545) values ranging from 1.425 to 3.713 with a mean value of 2.51, which corresponds to the premalignant range in our ML algorithm (Fig. 6).

Previously, various techniques based on optical spectroscopy and imaging have been evaluated to detect malignancies in the oral cavity.^46^^–^^49^ Cals et al.^50^ investigated the application of Raman spectroscopy for the intraoperative assessment of tumor margins in the prognosis of oral SCC. Linear discriminant analysis was used as a diagnostic algorithm to discriminate against healthy tissue types and oral SCC. One of the disadvantages of Raman spectroscopy is that the Raman effect is an extremely weak process in which 1 in $10^{6}$ to $10^{8}$ photons are Raman scattered.^51^ This weak phenomenon makes it extremely difficult to obtain a high-quality spectrum with the decreased integration times required for *in vivo* applications. Also, Raman spectroscopic approach requires high excitation power, extremely narrow bandwidths, and bulky and expensive instrumentation.^52^^,^^53^ Recently, a multimodal endomicroscopy incorporating hyperspectral and confocal imaging using a single foveated objective was developed and tested on *ex vivo* oral cancer samples.^54^ However, details regarding the diagnostic accuracy were not presented in the study. In the present study, we implemented a simple ratio-metric approach on the diffusely reflected light using the image intensity ratio R610/R545 that maps changes in oxygenated hemoglobin absorption in tissue for the detection of tumors in oral malignancies. The findings of the present study indicate a strong association between the increase in the red/green DR image ratio (R610/R545) and different types of oral precancers. With the use of site-specific algorithms and larger data sets, the diagnostic accuracy of the algorithms used for detection of OPMLs of the oral cavity would improve over time.

Furthermore, we believe that utilization of an ML algorithm provides a more reliable assessment of the cancer grade such that screening and diagnosis of cancer at the POC in real-time would become a reality. Additionally, the system incorporates fluorescence excitation at 405 nm, which can be used as an additional tool for locating the tumor margins. Previous studies have established marked differences in the fluorescence emission profile with 400- to 410-nm excitation and were able to correctly identify 20 out of 22 samples investigated.^55^ The increase in the emission intensity at wavelengths greater than 600 nm noticed in this study can be credited to PpIX, which is accumulated in cancer cells owing to the reduced activity of the ferrochelatase enzyme in cancer cells.^20^ In yet another study, an optical imaging system with excitation at 405 nm was implemented for detection of precancerous lesions in the oral cavity.^56^ The changes associated with tissues during progression of neoplasia at this excitation wavelength include neovascularization in the stromal region and loss of auto-fluorescence due to the breakdown of collagen matrices. The use of optical fluorescence imaging or staining with toluidine blue may increase the number of lesions detected compared with oral visual examination alone and may increase border distinction at a subjective level.^57^ This technique will also be helpful in patients who are reluctant to have biopsy but are anxious to know the stage of their lesion. Overall, the results of this study indicate that the wide-field multimodal imaging incorporating fluorescence and DR will be of great significance in the screening of oral malignancies at the community level.

## 5 Conclusion

The storage of data on a single cloud platform has multiple benefits. In addition to providing data safety, security, and ease of access to the screening centers, it facilitates compilation of screening data from different centers into an ML algorithm that provides real-time feedback to the device or the caregiver during the screening process. We believe that quantitative information on tissue status made available by the BMIS at the POC would lead to improved prognosis and patient management.

Different anatomical sites such as buccal mucosa, vermillion border of the lip, lateral and dorsal tongue, upper palate, and gingiva have different tissue structures, morphology, and reflectance characteristics. The light penetration and DR properties of various anatomical sites is different and could alter the sensitivity and specificity for detecting cancer at these sites. Therefore, site-specific algorithms, instead of one that incorporates data from all sites, will generate a higher diagnostic accuracy for screening of OPMLs. This has been validated in the case of patients under different stages of cancer in the buccal mucosa.

Although the BMIS was used to screen the oral cavity of around 350 people through this multicentric clinical trial, the clinical data presented relate to cases in which a biopsy was performed. In most cases in which a biopsy is not warranted or is ethically non-essential, the patients were advised to withdraw from their habits of pan or tobacco use and maintain oral hygiene. Since only two LEDs were used for each of the four wavelengths of light in the present design, uniformity of illumination was a major concern when the camera was to be used intraorally at short working distances from the tissue. Owing to this, the captured images were overexposed at some parts of the image, and the image data had to be discarded, despite repeated measurements. This was overcome by use of smaller form factor LEDs in a redesigned version of BMIS, currently being marketed by Sascan Meditech as OralScan^®^. Based on user feedback, the GUI of proprietary software and its features were upgraded in this version of the device to provide an improved user experience. Since we are using an ML algorithm, more and more data are collated into the algorithm with the increasing number of users. We hope that in time we will be able to evolve site-specific algorithms and facilitate early detection of oral precancers with improved accuracy. We believe that only through mass screening and early detection of OMPLs of the oral cavity will it be possible to lower the high rates of mortality associated with the disease. The ability of the device to guide the clinician to the most malignant site in a lesion for tissue biopsy will minimize false negatives, unnecessary multiple biopsies, and unwanted treatments, leading to lower costs for disease management and reduced patient trauma.

## Code, Data, and Materials Availability

The authors declare full availability of all data generated during the study and all material used for the analysis. Proprietary software was developed and used for operation of the device and data capture and analysis. No software code was associated with the main claims of the article. Table S1 in the Supplementary Material provides a list of all primary, processed, and pathological data used in the algorithm, and Table S2 in the Supplementary Material compares the diagnostic accuracies of the R610/R545 and R545/R575 ratios for discrimination of OPML and leukoplakia lesions from adjoining normal tissues of patients.
