# Supplementary material for: Bimodal multispectral imaging system with cloud-based machine learning algorithm for real-time screening and detection of oral potentially malignant lesions and biopsy guidance
Source: J Biomed Opt. 2021 Aug 16;26(8):086003. doi: 10.1117/1.JBO.26.8.086003 (PMC8367825; doi:10.1117/1.JBO.26.8.086003)
Supplement: Supplementary file 1 [file JBO_026_086003_SD001.pdf]

Supplementary Information:

The absorption spectra of Hb and HbO_2 displays isosbestic points at various spectral regions including the 540-550 nm range, where HbO_2 has a strong absorption (Figure S1). However, in the 610-650 nm range their absorption cross sections have maximum variance with HbO2 showing very lowest molar extinction coefficient. Thus, there is a strong rational to use the ratio of diffuse reflectance intensity at 610 and 545 nm for mapping of HbO2 absorption changes during early stages of cancer development in squamous epithelial tissues.

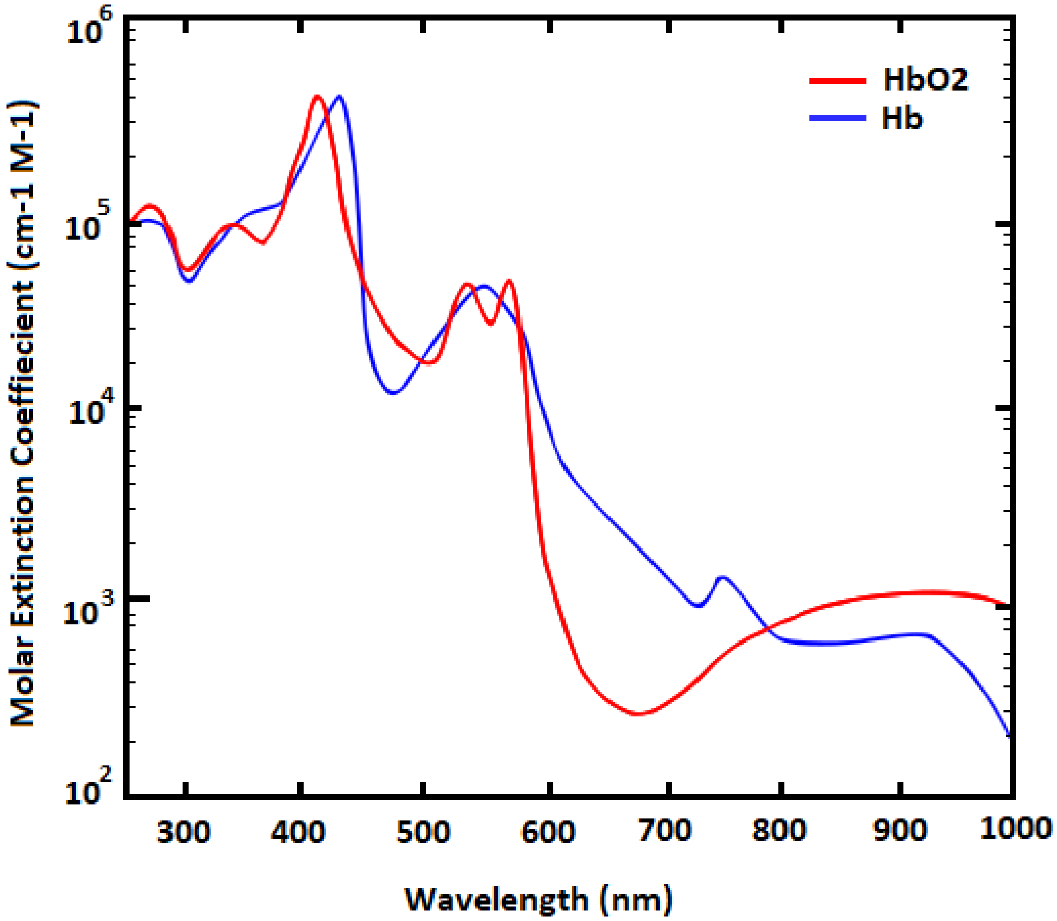

Figure S1. Molar extinction coefficient of HbO2 and Hb in the visible and near infrared wavelength range (adapted from Wikimedia Commons)

Supplementary Tables

Table S1: Clinical and pathological data from all the cases involving a guided biopsy, with the corresponding DR ratio (R610/R545) at the biopsy site and BMIS inference, which is based on the means and median in the study (<2-Normal, 2-3.5- Potentially malignant, >3.5- Malignant).

Sample No	Intraoral site	Clinical observations	Histopathology	DR Ratio	BMIS Inference
1	Buccal Mucosa	Patch, Non scrapable, 1*1cm, Oval, Whitish, Sessile, Fixed, Non tender	Mild Dysplasia	3.63	Low grade Malignant
2	Buccal Mucosa	Leukoplakia	Mild dysplasia	2.629	Potentially malignant
3	Buccal Mucosa	Patch and growth is present, which is 0.5*0.7cm and is irregular	Moderate Dysplasia	2.542	Potentially malignant
4	Alveolus Lower	Patch, Non scrapable, 1*1cm, Oval, Whitish, Sessile, Fixed, Fir, Non tender	Mild Dysplasia	3.63	Low grade Malignant
5	Buccal Mucosa	Leukoplakia	Moderate dysplasia	3.179	Potentially malignant
6	Buccal Mucosa	Leukoplakia	Mild dysplasia	3.147	Potentially malignant
7	Buccal Mucosa	Leukoplakia	Mild dysplasia	3.482	Potentially malignant
8	Buccal Mucosa	patch on right BM, 1cm away from the commissure of lip	Moderate Dysplasia	3.068	Potentially malignant
9	Inner Lip	Ulcer present in inter-junctional labial mucosa and vestibule -42 - 44 and gingiva -45, Ulcer 2*2cm irregular, reddish, firm fixed and tender	Mild dysplasia	3.6	Malignant
10	Buccal Mucosa	Growth verrucous -2*2.5cm in size, irregular, white in color.	Mild Dysplasia	2.312	Potentially malignant
11	Buccal Mucosa	OSMF -6 months, Erythematous surface 0.5*1cm size, irregular, toluidine blue test positive	Carcinoma in Situ (CIS)	2.602	Potentially malignant
12	Buccal Mucosa	White lesion	Hyperplasia	1.935	Border line Normal
13	Buccal Mucosa	Leukoplakia	Mild dysplasia	2.847	Potentially malignant
14	Buccal Mucosa	Leukoplakia	Hyperplasia	2.491	Potentially malignant
15	Buccal Mucosa	Leukoplakia	Mild dysplasia	3.113	Potentially malignant
16	Buccal Mucosa	Speckled Leukoplakia	Moderate Dysplasia	3.163	Potentially malignant
17	Dorsal Tongue	OSMF, Leukoplakia	Mild Dysplasia	3.126	Potentially malignant
18	Buccal Mucosa	Homogeneous leukoplakia	Severe Dysplasia	3.953	Malignant
19	Buccal Mucosa	Leukoplakia, traumatic fibrosis	Mild Dysplasia	3.859	Malignant

20	Buccal Mucosa	soft tissue greyish white in colour with underlying light brown areas. Hard in consistency	Hyperplasia	2.303	Potentially malignant
21	Floor of the Mouth	Verrucous CA	Mild Dysplasia	2.405	Potentially malignant
22	Alveolus Lower	CA Alveolus	Moderate Dysplasia	2.595	Potentially malignant
23	Lateral Tongue	Leukoplakia	Mild Dysplasia	2.639	Potentially malignant
24	Hard Palate	Verrucous hyperplasia	Mild Dysplasia	2.457	Potentially malignant
25	Lateral Tongue	Greyish white structure	Fibroepithelial polyp tongue	3.247	Potentially malignant
26	Buccal Mucosa	Stratified, squamous keratinized epithelium with dysplastic changes	Mild Dysplasia	3.744	Malignant
27	Gingiva	Stratified, squamous. Keratinized epithelium with dysplastic changes	Mild Dysplasia	4.115	Malignant
28	Tongue	White lesion	Lichen planus	2.276	Potentially malignant
29	Alveolus Lower	White soft tissue lesion	Ulcerated squamous mucosa with acanthosis and papilomatosis, subepithelial mild inflammation	2.633	Potentially malignant
30	Dorsal Tongue	Grey white tissue	Hyperplasia	4.689	Malignant
31	Buccal Mucosa	inflammatory cell	Erosive Lichen planus	2.546	Potentially malignant
32	Buccal Mucosa	Tan brown soft tissue	Hyperplasia	3.052	Potentially malignant
33	Buccal Mucosa	Tan brown soft tissue	Hyperplasia	3.025	Potentially malignant
34	Buccal Mucosa	Multiple tan brown soft tissue pieces collectively measuring 0.4*0.2cm.	Mild Dysplasia	2.222	Potentially malignant
35	Buccal Mucosa	Leukoplakia	Stratified squamous epithelium with parakeratosis and acanthosis	3.398	Potentially malignant
36	Buccal Mucosa	Ulceration over left retromolar	Mild Dysplasia	2.329	Potentially malignant
37	Lateral Tongue	Proliferative Growth	Pseudoepitheli o-matous hyperplasia with inflammation	4.166	Malignant

38	Buccal Mucosa	White lesion	Hyperplasia	2.725	Potentially malignant
39	Buccal Mucosa	Tan white soft tissue, 0.6*0.4cm	Moderate Dysplasia	2.356	Potentially malignant
40	Buccal Mucosa	Leukoplakia	Mild Dysplasia	4.104	Malignant
41	Hard Palate	Swelling which is fluctuant and tender	WDSCC	3.137	Potentially malignant
42	Lateral Tongue	Ulcer which is 3*3cm in size, firm in consistency, not fixed to underlying structures and non-tender	WDSCC	2.307	Potentially malignant
43	Buccal Mucosa	Ulceroproliferative lesion	WDSCC	3.459	High grade potentially malignant
44	Lateral Tongue	Ulcer	WDSCC	2.307	Potentially malignant
45	Buccal Mucosa	Ulcer in right BM - 36 and 16 extending posteriorly. 3*3cm, ulcerative, bleeds on touch. Oral submucous fibrosis - grade II mouth opening less than 10mm	PDSCC	5.844	High grade malignant
46	Buccal Mucosa	Ulcer Measuring 4cm, oval, pale, red, firm, not fixed to underlying structures, tender.	WDSCC	3.602	Malignant
47	Lateral Tongue	Ulcer which is 32*3cm irregular pale yellow and non-tender	MDSCC	3.852	Malignant
48	Lateral Tongue	Indurated ulcer	MDSCC	3.895	Malignant
49	Buccal Mucosa	Ulcer	WDSCC	4.63	Malignant
50	Buccal Mucosa	Ulcer, measuring about 3*4cm Oval reddish pink hard, fixed, tender	WDSCC	3.852	Malignant
51	Buccal Mucosa	Patch measuring 2*3cm, whitish, fixed, tender, mucosal thickening is present in right buccal mucosa with pus, submandibular lymph node is palpable	MDSCC	4.63	Malignant
52	Lateral Tongue	Ulcer, 3*3cm in size, pink in color, hard in consistency, fixed too underlying structures and tender on palpation	MDSCC	6.692	High grade malignant
53	Hard Palate	Ulcer	WDSCC	3.59	Malignant
54	Lateral Tongue	Ulcer measuring 3*3cm round whitish hard in consistency, fixed and nontender	MDSCC	4.181	Malignant
55	Lateral Tongue	Ulcer measuring 3*2cm size, oval, res in color, soft in consistency and tender	WDSCC	3.698	Malignant
56	Lateral Tongue	Growth at lateral border of tongue(44-46region), measuring about 2*2cm, soft in consistency,	WDSCC	3.773	Malignant

		sessile, fixed, non- tender, Grossly decayed 48			
57	Lateral Tongue	Whitish ulcerated non separable lesion in relation to 36, oval in shape with size 2*3cm. Lesion is soft, sessile, fixed and tender due to sharp tooth	WDSCC	2.706	Potentially malignant
58	Lateral Tongue	Swelling is gradual in onset, moderate rate of growth and its duration is 9 months, Swelling is 2*2cm, elliptical, whitish pink	WDSCC	3.379	Malignant
59	Buccal Mucosa	Ulcer present on BM extending from 45 till the retromolar area. The lesion is erythematous, firm, and tender. Right submandibular lymph node is round and fixed	MDSCC	3.301	Potentially malignant
60	Lateral Tongue	Size of ulcer, 3*4cm, oval in shape, sessile, soft, not fixed to underlying structure	PDSCC	5.034	High grade Malignant
61	Lateral Tongue	Ulcer present on left lateral border of tongue, 1.5*4cm size.	WDSCC	3.111	Potentially malignant
62	Alveolus Lower	Growth in pale pink and oval in shape, tender and sudden in onset, OSMF	PDSCC	3.82	Malignant
63	Alveolus Lower	Ulcerated lesion	PDSCC	2.247	Potentially malignant
64	Alveolus Lower	CA alveolus	MDSCC	2.132	Potentially malignant
65	Alveolus Upper	Carcinoma alveolus	MDSCC	2.368	Potentially malignant
66	Buccal Mucosa	Carcinoma BM	WDSCC	2.72	Potentially malignant
67	Alveolus Lower	Growth right lower alveolus verrucous carcinoma	MDSCC	2.306	Potentially malignant
68	Lateral Tongue	Ulcer border of tongue	WDSCC	2.995	Potentially malignant
69	Gingiva	Ulcer, oval in shape, red, soft and tender	WDSCC	3.267	Potentially malignant
70	Buccal Mucosa	Ulceroproliferative growth in left mandibular corner.4*3cm and present in gingivo buccal sulcus extending to retromolar area and buccal mucosa	MDSCC	3.497	High grade Potentially malignant
71	Hard Palate	Ulceroexophytic growth over hard palate	WDSCC	3.763	Malignant
72	Lateral Tongue	Ulcer tongue	PDSCC	3.485	High grade potentially malignant
73	Alveolus Upper	Ulcer	WDSCC	3.52	Malignant
74	Buccal Mucosa	Ulcerative lesion on BMR	MDSCC	4.875	Malignant

75	Buccal Mucosa	Erthematous swelling BML	WDSCC	3.238	Potentially malignant
76	Lateral Tongue	Non healing ulcer-right lateral border of tongue	WDSCC	8.189	High grade Malignant
77	Buccal Mucosa	Ulceroprofliferative growth in BML	MDSCC	4.218	Malignant
78	Buccal Mucosa	Malignant ulcer rt posterior BM	WDSCC	3.862	Malignant
79	Buccal Mucosa	Recurrent growth over left retromolar region	PDSCC	2.952	High grade potentially malignant
80	Buccal Mucosa	Ulcerated lesion over BML	WDSCC	3.589	Malignant
81	Lateral Tongue	Rd. multiple linear tissue,1.5*1*0.2cm	MDSCC	4.42	Malignant
82	Lateral Tongue	Ulcer	WDSCC	2.604	Potentially malignant
83	Alveolus Lower	Ulcerated lesion over upper alveolus	WDSCC	2.804	Potentially malignant
84	Dorsal Tongue	Exophytic growth tongue lesion	WDSCC	3.598	Malignant
85	Dorsal Tongue	Ulcer	WDSCC	6.564	Malignant
86	InnerLip	Mucosa covered tan brown soft tissue	WDSCC	4.345	Malignant
87	Alveolus Upper	white exophyte growth over left upper alveolus	WDSCC	4.107	Malignant
88	Lateral Tongue	Thick whitish patches posterior aspect of left border of tongue	PDSCC	4.197	Malignant
89	Buccal Mucosa	Ulcer	MDSCC	4.03	Malignant

Abbreviations used: WDSCC- well differentiated squamous cell carcinoma, MDSCC- moderately differentiated squamous cell carcinoma, PDSCC-poorly differentiated squamous cell carcinoma.

Table S2: Comparison of the diagnostic accuracy of R610/R545 ratio with R545/R575 ratio for discrimination of OPML and Leukoplakia lesions from adjoining normal tissues of patients

Diagnostic Accuracy	Patient Normal Vs OPML		Patient Normal Vs Leukoplakia	
	R610/R545	R545/R575	R610/R545	R545/R575
Sensitivity (%)	97.5	37.5	85.7	57.1
Specificity (%)	92.5	86.21	100	71.43
PPV	0.929	0.556	1	0.667
NPV	0.974	0.75	0.875	0.625
Cut-off Value	2.028	1.561	2.173	1.339
Sample Size	40	40	14	14
